# Expression of Genes Encoding Receptors for Classical Neurotransmitters, Neuropeptides and Hormones in the Substantia Nigra, Especially in Dopaminergic Neurons, in Intact Mice and Mouse Models of Parkinson’s Disease

**DOI:** 10.3390/cells14191570

**Published:** 2025-10-09

**Authors:** Dmitry Troshev, Ekaterina Pavlova, Vsevolod Bogdanov, Michael Ugrumov

**Affiliations:** Laboratory of Neural and Neuroendocrine Regulations, Koltzov Institute of Developmental Biology of the Russian Academy of Sciences, 119334 Moscow, Russia; dmitry.vad.troshev@gmail.com (D.T.); guchia@gmail.com (E.P.); vse-bogd@yandex.ru (V.B.)

**Keywords:** Parkinson’s disease, mouse model, substantia nigra pars compacta, dopaminergic neurons, fluorescence-activated cell sorting, receptor gene expression

## Abstract

Parkinson’s disease (PD) is characterized by degeneration of nigrostriatal dopaminergic neurons (DNs) and movement disorders. Low efficiency of pharmacotherapy requires improvement, e.g., using receptor agonists or antagonists as drugs. Our work aims to initiate these developments by studying the expression levels of genes encoding neurotransmitters, neuropeptides and hormone receptors in substantia nigra pars compacta (SNpc) cells and in isolated DNs in intact mice, and changes in expression of these genes in MPTP mouse models of PD at preclinical and clinical stages. Expression of all 12 studied genes was detected in the SNpc and only 10 in DNs—*Cckar* and *Glp1r* were undetectable. In intact mice, the expression of *Drd2*, *Grin2b*, *Grm1* and *Ntsr2* predominates in SNpc tissue, whereas that of *Gria2*, *Chrnb2*, *Gper1*, *Igf1r* is higher in DNs. In PD models, change in receptor gene expression was detected in DNs but not in SNpc tissue. In the preclinical PD, *Drd2* expression increased and *Gria2* decreased, whereas in a clinical model, *Drd2*, *Grm1*, *Ntsr2* expression decreased. Thus, the above genes are expressed in DNs and other cells of SNpc; expression of some genes changes in PD models, which opens up prospects for development of therapy using receptor agonists and antagonists.

## 1. Introduction

Parkinson’s disease (PD) is an incurable neurodegenerative disorder. It causes the progressive degradation of the nigrostriatal dopaminergic system involved in the regulation of motor function [1]. Approximately 10% of patients suffer from the monogenic form of the disease, caused by mutations in the *PARK2*, *PARK7*, *PINK1*, *ATP13A2*, *SYNJ1*, *SNCA*, *LRRK2*, and *VPS35* genes. Approximately 90% of patients have a polygenic (sporadic, idiopathic) form of the PD. Its development depends on the expression of risk genes under the influence of epigenetic factors in the external and internal environment [2,3].

The effectiveness of current symptomatic therapy using L-3,4-dihydroxyphenylalanine, an immediate precursor of dopamine, and dopamine receptor agonists that penetrate the blood–brain barrier is rather limited. This is due to the late diagnostics of PD based on the appearance of motor symptoms, so treatment begins only decades after the onset of neurodegeneration. By this point, there is a loss of 50–60% of dopaminergic neurons (DNs) in the substantia nigra pars compacta (SNpc) and 70–80% of dopamine level in the striatum [1]. This highlights the need to improve pharmacotherapy, mainly by enhancing the functional activity of surviving DNs and slowing their degeneration. Such therapy at the preclinical stage of PD will allow increasing the asymptomatic period of Parkinson’s disease and, consequently, the period of normal physical and social activity.

According to the translational medicine paradigm, the development of advanced and the improvement of current approaches to treating PD should be based on fundamental knowledge about the molecular mechanisms of neurodegeneration and neuroplasticity, primarily in the nigrostriatal system [3]. The most important insights into these mechanisms can be obtained by studying the pathogenesis of PD in patients. However, research in patients at the clinical stage of PD is highly limited and is impossible at the preclinical stage due to the lack of early PD diagnosis. Therefore, elucidating the mechanisms of PD pathogenesis and identifying new drug targets are mainly conducted using animal models of the disease [4,5].

Stimulation of the functional activity of the nigrostriatal pathway in PD patients and animal models of PD using dopamine precursors or agonists, on one hand, compensates for the dopamine deficiency in the striatum, but, on the other hand, can provoke neuronal death and dyskinesia [6,7]. Therefore, from this point on, traditional pharmacotherapy can no longer be used, and patients are treated with stereotactic surgery using high-frequency stimulation of the basal ganglia [8,9]. Long-term attempts to develop a cell technology to compensate for the dopamine deficiency in the striatum of PD patients have been unsuccessful [10,11]. Since invasive brain surgery is traumatic and can be associated with negative side effects, it is advisable to continue improving pharmacotherapy using fundamentally new approaches. One of them could be the use of nigrostriatal DNs receptor agonists or antagonists of receptors. The promise of this approach has recently been proven in the treatment of Alzheimer’s disease [12]. The development of such therapy of PD should be initiated by testing whether the development for PD is associated with a change in gene expression of receptors in the SNpc and DNs, in particular.

In this context, the most promising are receptors for physiologically active substances—classical neurotransmitters, neuropeptides and hormones, which are expressed in the DNs of the SNpc and participate in the regulation of the functional and metabolic activity, as well as the survival of these neurons [6,13,14,15,16].

Proceeding from the above, the main objective of this study was to evaluate expression of genes encoding receptors for classical neurotransmitters, neuropeptides and hormones in individual DNs of the SNpc in intact mice and in mouse neurotoxic models of the preclinical and clinical stages of PD. Using these data for further development of receptor agonist and antagonist therapy, it should be taken into account that these receptors are expressed in the SNpc tissue not only in DNs but also in other cells (glial, endothelial), possibly to a greater extent. Therefore, the second objective of this work was to evaluate the expression of genes for classical neurotransmitters, neuropeptides and hormones in the SNpc tissue in intact mice and in mouse models of PD at the clinical and preclinical stages. The third objective was to identify genes whose expression in SNpc tissue differs from that in isolated DNs. This would show that some genes for receptors are expressed in the SNpc in non-DNs.

## 2. Materials and Methods

### 2.1. Animals and Experimental Procedures

The study was conducted on male C57BL/6 mice (n = 47) aged 8–12 weeks and weighing 20–25 g, sourced from the Stolbovaya breeding center (SCBMT RAMS, Stolbovaya, Moscow region, Russia). The animals were housed in a vivarium under a 12 h light/dark cycle, at a temperature of 22 ± 1 °C, with ad libitum access to food and water.

Intact mice (n = 7) were used to compare receptor gene expression in SNpc tissue and in sorted DNs of the SNpc. The preclinical and clinical stages of PD were modeled in the mice according to a previously established protocol [17]. In the preclinical PD model, animals (n = 10) received subcutaneous injections of 1-methyl-4-phenyl-1,2,3,6-tetrahydropyridine (MPTP, Sigma-Aldrich, St. Louis, MO, USA) at doses of 8 and 10 mg/kg with a 24 h interval between injections (this model is hereafter referred to as 8–10 mg/kg MPTP). To model the clinical stage of PD, mice (n = 10) were administered MPTP in consecutively increasing doses of 8, 10, 12, 16, 20, 26, and 40 mg/kg with a 24 h interval between injections (this model is hereafter referred to as 8–40 mg/kg MPTP). In each control group, mice (n = 10 per group) were subcutaneously injected with saline (0.9% NaCl) following the same schedules as the MPTP groups. The material for analysis was obtained 24 h after the last injection of MPTP or saline.

This research was performed in full compliance with the National Institutes of Health Guide for the Care and Use of Laboratory Animals (8th edition, 2011) and received approval from the Animal Care and Use Committee of the Koltzov Institute of Developmental Biology of the Russian Academy of Sciences (Protocol No. 75, dated 2 November 2023). The experimental design and reporting adhere to the ARRIVE guidelines.

### 2.2. Dissection of the Substantia Nigra Pars Compacta, Preparing a Cell Suspension, and Staining Dopaminergic Neurons

The SNpc was dissected using our previously developed protocol with slight modifications [18]. Intact mice (n = 7) along with animals from experimental and control groups (n = 10 per group) were subjected to isoflurane anesthesia (gas flow: 250 mL/min; concentration: 2.4%) followed by decapitation and brain extraction. Serial 200 μm thick coronal sections containing the SNpc were prepared from each brain using a Leica VT1200S vibratome (Leica Biosystems, Wetzlar, Germany) according to the mouse brain stereotaxic atlas [19], from −2.54 mm to −3.88 mm caudal to bregma. SNpc samples from one hemisphere of individual sections were immediately frozen in liquid nitrogen and stored at −70 °C for subsequent RNA extraction. Complementary SNpc tissue from the contralateral hemisphere of the same sections, along with tissue from both hemispheres of additional sections, underwent enzymatic digestion. This material was incubated for 30 min at 37 °C under constant agitation in 2 mg/mL papain solution (Sigma-Aldrich, Saint Louis, MO, USA) prepared in DMEM (Gibco, Thermo Fisher Scientific, Waltham, MA, USA). Enzymatic activity was terminated by adding 4 °C-chilled fetal bovine serum (Gibco, Thermo Fisher Scientific, Waltham, MA, USA) to achieve a final concentration of 10% (*v*/*v*). The samples were subsequently centrifuged at 70× *g* for 30 s. Following supernatant removal, the pelleted SNpc sections underwent two washings using Hank’s Balanced Salt Solution (Gibco, Thermo Fisher Scientific, Waltham, MA, USA) and were centrifuged after each washing as mentioned above.

For DNs identification, SNpc sections were stained using 10 µM DRAQ5 nuclear dye (Abcam, Cambridge, UK) and 50 nM GBR-BODIPY FL (synthesized by the IBCh RAS Oxylipin Laboratory) according to published methodology [18]. Throughout all preparation and staining procedures for preparing SNpc cell suspensions and DNs labeling, 100 U/mL RiboLock RNase Inhibitor (Thermo Fisher Scientific, Waltham, MA, USA) was maintained in all solutions to preserve RNA integrity.

### 2.3. Fluorescence-Activated Cell Sorting

DNs labeled with DRAQ5 and GBR-BODIPY FL were isolated using a FACSAria III cell sorter (BD, Franklin Lakes, NJ, USA) configured with a 100 μm nozzle at a pressure of 20 psi. Thresholds for DRAQ5 and GBR-BODIPY FL fluorescence were established by analyzing an unstained cell suspension. The DRAQ5 fluorophore was excited using a 640 nm laser with emission captured through a 670/30 nm bandpass filter (DRAQ5 channel). Similarly, GBR-BODIPY FL excitation was achieved with a 488 nm laser and emission detected through a 530/30 nm bandpass filter (GBR-BODIPY FL channel). Sorted DNs were collected into tubes prefilled with 250 μL of TRI-reagent (Sigma-Aldrich, Saint Louis, MO, USA). Immediately following sorting, tubes were vigorously vortexed, frozen in liquid nitrogen, and maintained at −70 °C until subsequent RNA extraction.

The distribution profiles of all recorded events (particles detected by the sorter) were analyzed through histogram generation using FCS Express 7 software (De Novo Software, Pasadena, CA, USA).

### 2.4. Real-Time Polymerase Chain Reaction

Upon thawing, SNpc tissue samples were homogenized in 1 mL of TRI-reagent (Sigma-Aldrich, Saint Louis, MO, USA) through vigorous pipetting. Similarly, thawed samples containing sorted DNs were supplemented with 750 μL of TRI-reagent. Total RNA extraction from both SNpc tissue and sorted DNs was performed following the manufacturer’s protocol for TRI-reagent. To enhance RNA precipitation efficiency, 1 μL of glycogen (Thermo Fisher Scientific, Waltham, MA, USA) was added to all samples. RNA concentration was quantified using a NanoDrop 8000 spectrophotometer (Thermo Fisher Scientific, Waltham, MA, USA). To remove genomic DNA contamination, total RNA was treated with DNase I, RNase-free. (Thermo Fisher Scientific, Waltham, MA, USA) according to the manufacturer’s specifications. Reverse transcription for SNpc-derived samples was performed using the MMLV RT kit (Evrogen, Moscow, Russia) with 0.25 μg RNA input per reaction. For samples obtained from sorted DNs, cDNA synthesis was carried out using the Maxima H Minus First Strand cDNA Synthesis Kit (Thermo Fisher Scientific, Waltham, MA, USA) with random hexamer primers, utilizing 0.15 μg of RNA per reaction. cDNA concentrations were determined using the NanoDrop 8000 spectrophotometer. Quantitative polymerase chain reaction (qPCR) analysis was conducted on a QuantStudio 12k Flex system (Applied Biosystems, Waltham, MA, USA) using qPCRmix-HS SYBR+LowROX (Evrogen, Moscow, Russia) and gene-specific oligonucleotide primers (Evrogen, Moscow, Russia) (Table 1), with 500 ng of cDNA template per reaction.

Gene expression analysis was performed utilizing the 2^−ΔΔCt^ method, normalizing the expression of receptor-encoding genes to the expression of *Cyc1*. *Cyc1* was chosen as an endogenous control based on the stability of its expression in a mouse model of PD, showing less variation in expression compared to *Rpl13* [20], which had previously been recommended as an optimal housekeeping gene for assessing gene expression in neurodegenerative diseases [21].

Results were calculated as geometric means for each experimental group [22] and expressed as fold-change values relative to SNpc tissue from either intact mice or control groups. The 2^−ΔΔCt^ values for SNpc tissue from intact mice or control groups were designated as the baseline value of 1 for comparative analysis.

### 2.5. Statistical Analysis

Statistical analysis was conducted using GraphPad Prism 9 software (GraphPad Software, San Diego, CA, USA). The normality of data distribution across groups was assessed using the Shapiro–Wilk test. Pairwise comparisons were performed using either the unpaired *t*-test for normally distributed data or the Mann–Whitney test for non-normally distributed data, based on the distribution characteristics. Results are expressed as mean ± standard error of the mean. A *p*-value ≤ 0.05 was considered statistically significant.

## 3. Results

### 3.1. Expression of Genes Encoding Neurotransmitter, Neuropeptide and Hormone Receptors in the Substantia Nigra Pars Compacta Tissue and in Sorted Dopaminergic Neurons of the Substantia Nigra Pars Compacta in Intact Mice

**Figure 1:**
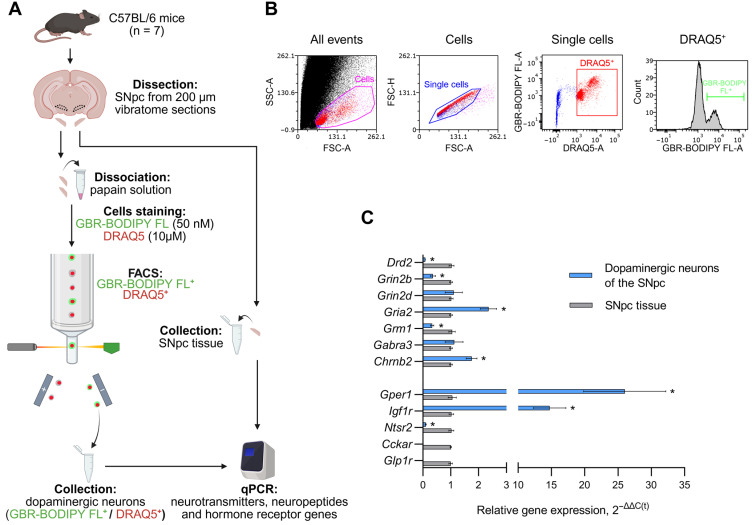
Expression of genes encoding receptors for neurotransmitters, neuropeptides, and hormones in substantia nigra pars compacta (SNpc) tissue and in sorted SNpc dopaminergic neurons in intact mice. (**A**) Schematic representation of experimental procedures. (**B**) Isolation of dopaminergic neurons used for fluorescence-activated cell sorting (FACS). (**C**) Expression of neurotransmitter receptor genes—*Drd2*, *Grin2b*, *Grin2d*, *Gria2*, *Grm1*, *Gabra3* and *Chrnb2*, as well as neuropeptide and hormone receptor genes *Gper1*, *Igf1r*, *Ntsr2*, *Cckar* and *Glp1r* in SNpc dopaminergic neurons and in SNpc tissue, their comparison. Differences were assessed by unpaired *t*-test or the Mann–Whitney test depending on the distribution type (* *p* ≤ 0.05 vs. SNpc tissue set as 1). DRAQ5^+^, DRAQ5-stained cells; FSC, forward scatter; GBR-BODIPY FL^+^, GBR-BODIPY FL-stained cells; qPCR, quantitative polymerase chain reaction; SSC, side scatter.

According to our data, expression of all 12 studied genes was detected in the SNpc tissue, whereas only 10 were expressed in isolated DNs. In fact, expression of *Cckar* and *Glp1r* was not detectable. We then compared the expression of the neurotransmitter receptor genes *Drd2*, *Grin2b*, *Grin2d*, *Gria2*, *Grm1*, *Gabra3*, and *Chrnb2*; the neuropeptide receptor gene *Ntsr2*; and the hormone receptor genes *Gper1* and *Igf1r* in sorted DNs with the expression of the same genes in SNpc tissue in intact mice (Figure 1A,B). It was shown that the expression of *Drd2* was 13.21-fold higher in the SNpc tissue compared with DNs (*p* < 0.0001) (Figure 1C). *Grin2b* and *Grm1* expression was 2.84-fold (*p* < 0.0001) and 3.16-fold (*p* = 0.0002) higher in SNpc tissue. Conversely, *Gria2* and *Chrnb2* expression was 2.34-fold (*p* = 0.0007) and 1.73-fold higher in DNs than in SNpc tissue. However, no differences were found in the expression of *Grin2d* and *Gabra3* in SNpc tissues and DNs. The expression of *Grep1* and *Igf1r* was higher in DNs than in SNpc tissue by 24.67 times (*p* = 0.0016) and 14.45 times (*p* = 0.0006), respectively (Figure 1C), whereas the expression of *Ntsr2* in SNpc tissue exceeded that in DNs by 10.28 times (*p* = 0.0006).

### 3.2. Expression of Genes Encoding Neurotransmitter, Neuropeptide and Hormone Receptors in the Substantia Nigra Pars Compacta Tissue and in Sorted Dopaminergic Neurons of the Substantia Nigra Pars Compacta in Mice When Modeling the Preclinical Stage of Parkinson’s Disease

**Figure 2:**
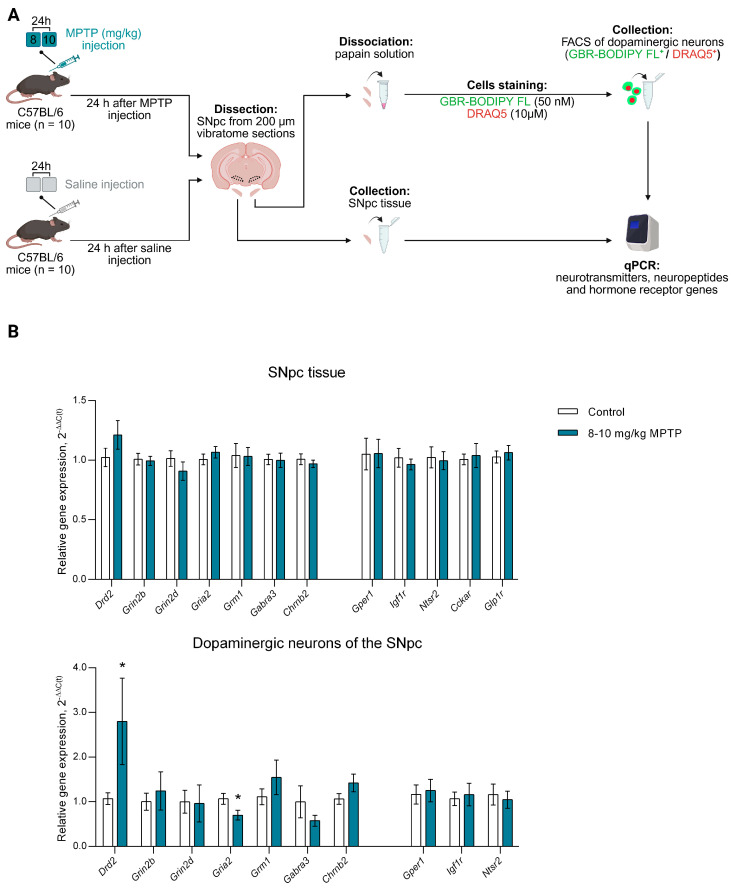
Expression of genes encoding receptors for neurotransmitters, neuropeptides and hormones in the substantia nigra pars compacta (SNpc) tissue and in sorted SNpc dopaminergic neurons in a mouse model of preclinical Parkinson’s disease (PD) and in a control group. (**A**) Schematic representation of the experimental procedures: the preclinical stage of PD was modeled in mice by subcutaneous injections of 1-methyl-4-phenyl-1,2,3,6-tetrahydropyridine (MPTP) at doses of 8 and 10 mg/kg with an interval of 24 h between injections, whereas in the control saline was administered along the same scheme. (**B**) Expression of neurotransmitter receptor genes *Drd2*, *Grin2b*, *Grin2d*, *Gria2*, *Grm1*, *Gabra3*, and *Chrnb2* and neuropeptide and hormone receptor genes *Gper1*, *Igf1r*, *Ntsr2*, *Cckar* and *Glp1r* in SNpc tissue and SNpc dopaminergic neurons in preclinical PD mice and controls. Differences were assessed by unpaired *t*-test (* *p* ≤ 0.05 vs. controls set as 1). DRAQ5^+^, DRAQ5-stained cells; FSC, forward scatter; GBR-BODIPY FL^+^, GBR- BODIPY FL-stained cells; qPCR, quantitative polymerase chain reaction.

When modeling the preclinical PD (8,10 mg/kg MPTP) (Figure 2A), the expression of neurotransmitter receptor genes *Drd2*, *Grin2b*, *Grin2d*, *Gria2*, *Grm1*, *Gabra3*, and *Chrnb2* did not change in the SNpc tissue compared to controls (Figure 2B). However, this was not always the case for gene expression in DNs of the SNpc. Indeed, *Drd2* expression in DNs of the SNpc increased 2.62-fold (*p* = 0.0446) and *Gria2* expression decreased 1.52-fold (*p* = 0.0415) in a mouse model of preclinical PD compared to controls. The expression of *Grin2b*, *Grin2d*, *Grm1*, *Gabra3*, *and Chrnb2* remained unchanged (Figure 2B).

Regarding the expression of neuropeptide and hormone receptors genes in a mouse preclinical model of PD, it was shown that the expression of *Gper1*, *Igf1r*, *Ntsr2*, *Cckar* and *Glp1r* in SNpc tissue and DNs of the SNpc was not changed compared to the control (Figure 2B).

### 3.3. Gene Expression of Neurotransmitter, Neuropeptide and Hormone Receptors in the Substantia Nigra Pars Compacta Tissue and in Sorted Dopaminergic Neurons of the Substantia Nigra Pars Compacta in Mice When Modeling the Clinical Stage of Parkinson’s Disease

**Figure 3:**
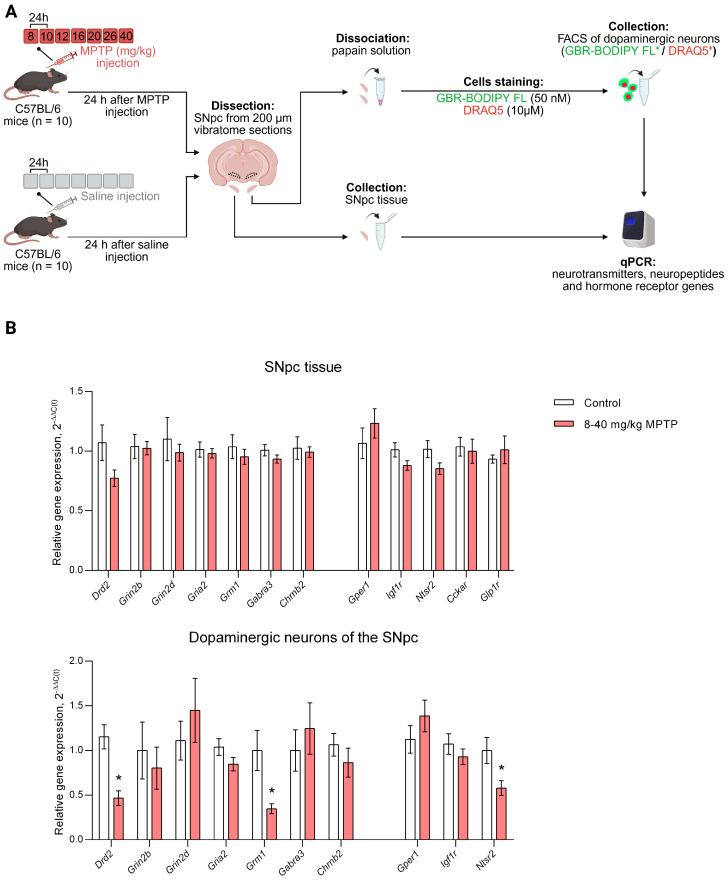
Expression of genes encoding receptors for neurotransmitters, neuropeptides and hormones in the substantia nigra pars compacta (SNpc) tissue and in sorted SNpc dopaminergic neurons in a mouse model of clinical Parkinson’s disease (PD) and in a control group. (**A**) Schematic representation of the experimental procedures: the clinical stage of PD was modeled in mice by subcutaneous injections of 1-methyl-4-phenyl-1,2,3,6-tetrahydropyridine (MPTP) at doses of 8, 10, 12, 16, 20, 26 and 40 mg/kg with an interval of 24 h between all the injections, whereas in the control saline was administered along the same scheme. (**B**) Expression of neurotransmitter receptor genes *Drd2*, *Grin2b*, *Grin2d*, *Gria2*, *Grm1*, *Gabra3*, and *Chrnb2* and neuropeptide and hormone receptor genes *Gper1*, *Igf1r*, *Ntsr2*, *Cckar* and *Glp1r* in SNpc tissue and SNpc dopaminergic neurons in clinical PD mice and controls. Differences were assessed by unpaired *t*-test (* *p* ≤ 0.05 vs. controls set as 1). DRAQ5^+^, DRAQ5-stained cells; FSC, forward scatter; GBR-BODIPY FL^+^, GBR- BODIPY FL-stained cells; qPCR, quantitative polymerase chain reaction.

When modeling the clinical stage of PD (8–40 mg/kg MPTP) (Figure 3A), it was shown that in the SNpc tissue, the expression of *Drd2*, *Grin2b*, *Grin2d*, *Gria2*, *Grm1*, *Gabra3*, and *Chrnb2* did not change (Figure 3B). However, in DNs of the SNpc in the PD clinical stage model, *Drd2* expression decreased by 2.46 fold (*p* = 0.0004) and *Grm1* expression decreased by 2.88 fold (*p* = 0.0095), while the expression of other genes remained at the control level (Figure 3B).

In the mouse model of clinical PD, no changes in the expression of the studied neuropeptide and hormone receptor genes were detected in SNpc tissue compared to controls. In DNs of the SNpc, the only change in the expression of the studied genes was a 1.73-fold decrease in *Ntsr2* expression (*p* = 0.0205) compared to the control (Figure 3B).

## 4. Discussion

According to the first objective of this work, it was shown that all 12 studied genes are expressed in the SNpc tissue, whereas only 10 genes were expressed in sorted DNs. In fact, DNs lack the expression of *Cckar* for cholecystokinin A receptor and *Glp1r* expression for glucagon-like peptide 1 receptor. To meet the second objective of this study, we compared the expression of genes encoding receptors for classical neurotransmitters, neuropeptides and hormones in SNpc tissue and precisely in DNs in intact mice. Since regulation of DNs of the SNpc is provided mainly by such classical neurotransmitters as dopamine, glutamate, gamma-aminobutyric acid (GABA) and acetylcholine [13], the gene expression of receptors for these neurotransmitters has been tested. Thus, it was shown that the expression of *Drd2*, encoding the D2 receptor, was more than 13 times higher in SNpc compared to DNs of the SNpc. Despite the fact that D2 autoreceptors play a key role in maintaining dopamine homeostasis, particularly in regulating dopamine release [23], it turned out that *Drd2* is expressed in DNs to a lesser extent than in other SNpc cells, where they are not autoreceptors. According to literature data, *Drd2* is expressed in the SNpc in astrocytes, and through these receptors dopamine modulates calcium activity—promoting changes in cytosolic Ca^2+^ levels [24], stimulating the synthesis of glial cell line-derived neurotrophic factor [25], and suppressing the production of angiotensinogen and its metabolite—angiotensin II, thereby preventing the development of neuroinflammation [26]. D2 receptors have also been found in microglia, where their activation appears to reduce neuroinflammation [26,27].

In addition to dopamine, glutamate plays an important role in the regulation of DNs of the SNpc, affecting ionotropic and metabotropic receptors. Therefore, we paid special attention to the expression of the genes for N-methyl-D-aspartate (NMDA) receptor, α-amino-3-hydroxy-5-methyl-4-isoxazolepropionic acid (AMPA) receptor, and glutamate metabotropic receptors (mGluRs), including the genes of their protein subunits. We also took into account that the properties of NMDA receptors are determined by their subunits [28]. Thus, NMDA receptors with the 2B subunit have a higher conductance at high concentrations of agonists compared to NMDA receptors with the 2D subunit [29]. In this regard, we assessed the expression of the genes encoding the 2B and 2D subunits of the NMDA receptor. It was shown that the expression of *Grin2b*, encoding the NMDA receptor subunit 2B, is lower in SNpc DNs than in the SNpc tissue. This is consistent with the fact that in DNs of the SNpc in rodents, the 2B subunit of the NMDA receptor is detected in trace amounts [30], whereas in primates it is not detectable [31]. At the same time, we did not find any differences in the expression of *Grin2d*, encoding NMDA receptor subunit 2D in DNs of the SNpc and SNpc tissue. Our data on the *Grin2b*/*Grin2d* expression ratio indicate that *Grin2d* expression predominates in DNs of the SNpc. Thus, we suggest that NMDA receptors in DNs of the SNpc consist predominantly of 2D subunits.

The specific patterns of gene expression of NMDA receptor subunits in intact mice that we found may be important for understanding the role of glutamate in the degeneration of DNs. Indeed, excessive accumulation of glutamate in the synaptic cleft activates NMDA receptors, inducing excitotoxicity in DNs of the SNpc [32,33,34]. On the other hand, the presence of the 2D subunit in the NMDA receptor leads to an attenuation of the Ca^2+^ current through this receptor [29], which reduces the risk of excitotoxicity.

Along with the NMDA receptors, AMPA receptors, which may include subunits 1, 2, 3, and 4, are involved in the regulation of DNs of the SNpc by glutamate [35]. Subunit 1 was found in 43% of DNs, subunits 2 and 3 were detected in the majority of such neurons, whereas subunit 4 was not detected at all [35]. Given that the permeability of AMPA receptors for monovalent and divalent cations depends on the presence of subunit 2 [36], we assessed the expression of *Gria2*, which encodes subunit 2. According to our data, the expression of *Gria2* is much higher in DNs of the SNpc than in SNpc tissue. Previous studies have shown not only that AMPA receptor subunit 2 is expressed in almost all DNs of the SNpc in mammals, but also that its mRNA content is higher in DNs of the SNpc than in DNs located in the ventral tegmental area [31,35].

In addition to NMDA and AMPA receptors, mGluRs, which are coupled via G protein to second messengers, are involved in the regulation of DNs of the SNpc [37]. Using in situ hybridization, it was shown that among all types of mGluRs, only mGluR type 1 is characteristic of the SNpc [38]. Stimulation of mGluR1 in the SNpc increases dopamine release in the striatum of freely moving rats [39]. Our results indicate that the expression of *Grm1*, encoding mGluR1, in DNs of the SNpc is lower than in the SNpc tissue. This is explained by the fact that among the 1a, 1b, 1c, 1d and 1g isoforms of mGluR1 formed as a result of alternative splicing, only isoform 1d is characteristic of DNs of the SNpc but is not the most widespread in the brain compared with isoform 1a and the other 3 isoforms of mGluR1 [37].

Inhibitory control of DNs located in the SNpc is mediated not only through D2 autoreceptors but also through GABA receptors. Regulation of DNs by GABA is the most extensive—at least 50–70% of the afferents to these neurons are GABAergic [40]. In addition, in vivo synaptic responses are mediated predominantly or exclusively via GABA_A_ receptors [41]. The latter is a heteropentameric chloride ion channel, which includes predominantly subunit alpha3 in DNs of the SNpc in humans and rodents [42,43]. Our results indicate that *Gabra3*, encoding GABA_A_ receptor subunit alpha3, is expressed to the same extent in DNs of the SNpc and in the SNpc tissue.

In addition to dopamine, glutamate, and GABA, acetylcholine contributes to the regulation of DNs in the SNpc via nicotinic acetylcholine receptors (nAChRs) [13,44]. Among the subunits that form nAChR, we studied the expression of *Chrnb2*, which encodes the β2 subunit. Previous studies using in situ hybridization and single-cell qPCR have shown that the β2 subunit gene is expressed in the SNpc both in DNs and GABAergic neurons [45,46]. Our results suggest that *Chrnb2* expression and probably synthesis of the β2 nAChR subunit predominate in DNs of the SNpc compared to other SNpc cells.

Along with classical neurotransmitters, neuropeptides and hormones contribute to the regulation of DNs located in the SNpc. Thus, neurotensin (NT), insulin-like growth factor 1 (IGF1), and estrogens are involved not only in the regulation of neurotransmission and metabolism of these neurons but also have a neuroprotective effect [15]. DNs of the SNpc have NT receptors of types 1, 2, and 3 [47,48], but in this work, we assessed the expression of *Ntsr2*, which encodes NT receptor type 2. Through this receptor, NT exerts its influence on the synaptic plasticity of DNs of the nigrostriatal system [49]. In this study, we demonstrated that *Ntsr2* expression in DNs of the SNpc is lower than in SNpc tissue. Previous studies using in situ hybridization have detected *Ntsr2* transcripts in the cell bodies of DNs and to a greater extent in their dendrites [50,51,52]. According to our data, *Ntsr2* is most probably expressed in glial cells surrounding DNs, as previously shown for the ventral tegmental area. In this brain region, *Ntsr2* is predominantly expressed in astrocytes [53]. Our data provide additional information on *Ntsr2* expression in the SNpc tissue, although the cellular localization of transcripts outside of DNs should be clarified in future studies using in situ hybridization.

According to our results, the expression of *Igf1r*, encoding the IGF1 hormone receptor, was more than 14-fold higher in DNs of the SNpc compared to SNpc tissue. The IGF1 receptor has been previously found in DNs of the SNpc [16,54]. IGF, affecting this receptor, regulates neuronal excitability and inhibits the release of dopamine from the dendrites and cell bodies of DNs [16]. In addition, IGF1 affects the signaling cascades triggered by estrogens. Thus, inhibition of the IGF1 receptor attenuates the neuroprotective effect of both estrogens and IGF1 on DNs of the SNpc in a PD model [55]. In turn, the antagonist G protein-coupled estrogen receptor 1 (GPER1) attenuates the anti-apoptotic effect of IGF1 when DNs are treated with the neurotoxin—MPTP [56]. In contrast to NT and IGF1 receptors, the localization of GPER1 in brain cells is unclear. It is only known that GPER1 is present in the SNpc [57], but it is unknown in which SNpc cells this receptor is expressed. In this context, one of our results is of particular interest. This is more than 24-fold prevalence of *Gper1* expression in DNs of the SNpc compared to SNpc tissue. The high expression of *Igf1r* and *Gper1* that we found in DNs of the SNpc indicates neuron-specific expression of these genes and suggests an important role for the IGF1 receptor and GPER1 in the regulation of DNs.

In contrast to most of the genes assessed in this study (see above), expression of the *Cckar* and *Glp1r* was detected only in the SNpc tissue, but not in DNs of the SNpc. This suggests that the neuroprotective effect of cholecystokinin and glucagon-like peptide-1 reported in previous studies [58,59] is mediated by non-neuronal, apparently glial cells.

According to the third objective, we assessed changes in the expression of genes of receptors involved in the regulation of DNs located in the SNpc using a subchronic mouse model of progressive degradation of the nigrostriatal dopaminergic system at the preclinical and clinical stages of PD [17]. The use of this model makes it possible to study neurodegeneration and compensatory mechanisms at each stage of PD.

**Figure 4:**
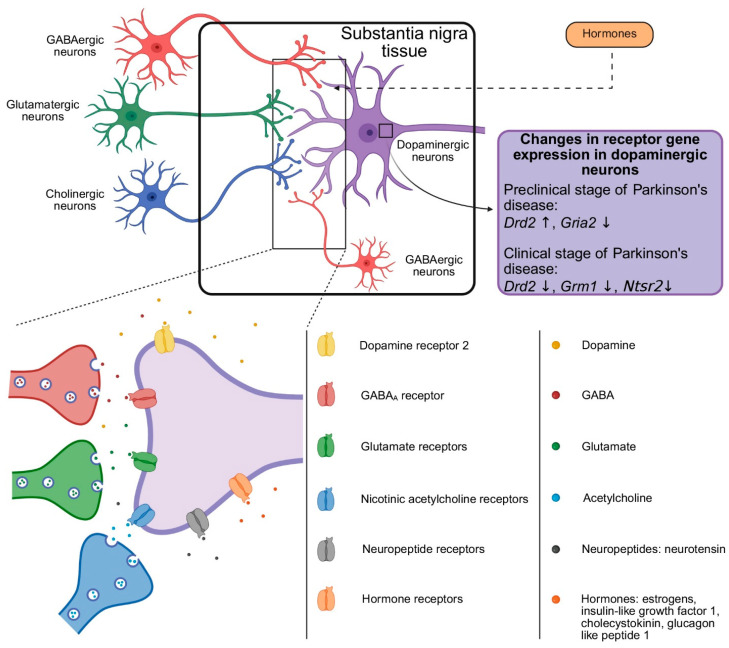
Schematic representation of receptor-mediated regulation of substantia nigra pars compacta dopaminergic neurons by neurotransmitters, neuropeptides and hormones with an indication of changes in the expression of neurotransmitter receptor genes (*Drd2*, *Gria2*, *Grm1*) and a neuropeptide gene (*Ntsr2*) in these neurons in mice at modeling preclinical and clinical stages of Parkinson’s disease. ↓, a decrease; ↑, an increase. GABA, gamma-aminobutyric acid.

We found a decrease in expression of *Gria2* encoding glutamate ionotropic receptor AMPA type subunit 2 in DNs of the SNpc in mice in a model of the preclinical stage of PD with reduced dopamine content in the striatum (Figure 4). This may be due to the adaptation of these neurons to glutamate receptor activation. Indeed, striatal dopamine deficiency leads to a decrease in the inhibitory effect of GABAergic neurons of the external capsule of the globus pallidus on neurons of the subthalamic nucleus. This, in turn, leads to an increase in the activity of glutamatergic neurons of the subthalamic nucleus, which have an excitatory influence on DNs of the SNpc [60]. One of the reasons for a change in the functioning of nigrostriatal DNs in PD is the disruption of the basal ganglia circuit functioning and not only the impairment of the pacemaker activity of DNs and an increase in their burst firing [61,62,63]. Therefore, it is logical to assume that a decrease in gene expression and synthesis of glutamate receptor subunits is aimed at preventing excitotoxicity in DNs of the SNpc and, hence, maintaining their normal functioning. This assumption is indirectly confirmed by a decrease in the number of neurons of the SNpc immunoreactive for glutamate ionotropic receptor AMPA type subunit 2 in rats following degradation of the nigrostriatal system [64].

It should be noted that synaptic regulation of nigrostriatal DNs by glutamate is mediated to a greater extent through NMDA receptors than through AMPA receptors [65]. Activation of NMDA receptors leads to excitotoxicity and degeneration of DNs located in the SNpc [32,33,66]. However, it should be borne in mind that there is an interaction between NMDA and AMPA receptors. Depolarization of the postsynaptic membrane caused by the AMPA receptor leads to the removal of Mg^2+^ through the NMDA receptor ion channel and the subsequent influx of calcium ions through the same receptor [67,68]. Based on the above, the decrease in the expression of *Gria2*, which we found in a mouse model of preclinical PD, may be important for the regulation of NMDA receptor activity and, hence, DNs of the SNpc.

In a mouse model of preclinical PD, we also found an increase in the expression of *Drd2* in DNs of the SNpc (Figure 4), which may be caused by an increase in dopamine release from these neurons. However, this assumption is in poor agreement with the literature data obtained in other models of preclinical PD. Thus, Good et al. (2011) showed that MitoPark mice with homozygous loss of mitochondrial transcription factor A have a decrease in dopamine release from the axons of DNs even before the onset of motor impairment [69]. A decrease in dopamine release was also shown in our previous study of the acute mouse MPTP model of preclinical PD using microdialysis [70]. Proceeding from the above, the increase in dopamine release from DNs of the SNpc cannot be explained by an increase in *Drd2* expression. On the other hand, D2 autoreceptors are known to regulate the spike frequency of DNs according to negative feedback through G-protein-coupled potassium channels [71]. In this context, we suggest that the increase in *Drd2* expression is a manifestation of the adaptation of nigrostriatal DNs to increased firing activity in the preclinical stage of the PD model and contributes to the decrease in this activity. The increase in *Drd2* expression may also contribute to the neuroprotection of DNs, since dopamine is known to affect mitochondrial function through D2 autoreceptors by inhibiting the intracellular cAMP/protein kinase A pathway [72].

The decrease in the expression of *Grm1* encoding mGluR1, which we found in DNs in a mouse model of PD clinical stage (Figure 4), may be a result of glutamate-induced hyperactivity of surviving DNs. Kaneda et al. came to a similar conclusion, suggesting that the down-regulation of mGluR1α in the SNpc, found in a monkey model of PD, exerts a compensatory reverse of the overactivity of the subthalamic nucleus-derived glutamatergic input [73]. It should be noted that changes in *Grm1* expression and mGluR1 content in the PD model are characteristic not only of nigrostriatal DNs but also of other neurons of the nigrostriatal system. Thus, it was shown with positron emission tomography in transgenic rats overexpressing the human α-synuclein gene that as degradation of the nigrostriatal dopaminergic system progresses, the content of striatal mGluR1 decreases [74].

Regulation of DNs is largely provided by dopamine via D2 autoreceptors. Indeed, when modeling in mice the preclinical stage of PD, we observed an increase in *Drd2* expression in DNs of the SNpc, whereas in the model of the clinical stage of PD, a decrease in *Drd2* expression was found (Figure 4). We assume that a decrease in *Drd2* expression in DNs of the SNpc may be accompanied by a decrease in the synthesis of D2 receptors and their content in the cytoplasmic membrane. This may result in an increase in the firing activity of surviving nigrostriatal DNs due to a decrease in the activation of G-protein-coupled potassium channels [71].

In addition to classical neurotransmitters, neuropeptides and hormones are involved in the regulation of nigrostriatal DNs. Thus, in a mouse model of the clinical stage of PD, we found a decrease in the expression of *Ntsr2*, the gene encoding NT receptor type 2, in DNs of the SNpc (Figure 4). These data are in good agreement with those obtained in patients with PD. Indeed, autoradiography showed a significant decrease in the radioactive ligand binding to NT receptors in the SNpc in patients with PD [75], while the NT content in the SNpc was increased [76].

Of particular interest is the simultaneous decrease in *Drd2* and *Ntsr2* expression, which we observed in a mouse model of clinical stage PD. It is known that the interaction of NT with the NT receptor type 2 in DNs of the SNpc causes long-term depression of D2 receptor-mediated inhibitory synaptic current [49]. In this context, the fact that long-term depression is caused by NT released from DNs located in the SNpc attracts special attention. This is necessary to maintain the excitability of these neurons with an increase in dopamine levels in the SNpc [77]. Based on the above, we assume that a decrease in *Ntsr2* expression is accompanied by a decrease in the synthesis of NT receptor type 2 and its content in the cytoplasmic membrane. In turn, this may lead to a decrease in long-term depression of D2 receptor-mediated inhibitory synaptic current in DNs of the SNpc and a leveling of their firing activity caused by a decrease in *Drd2* expression.

Meanwhile, we have not detected any changes in the expression of receptor genes in the SNpc tissue either in the preclinical PD model or in the clinical PD model, although such changes cannot be ruled out. This is probably due to the fact that we have obtained an integral characteristic of the expression of individual genes in different SNpc cells. With multidirectional changes in the expression of such genes, the integral indicator could be minimized or even leveled. This means that the study of gene expression in a single cell population in health and pathology is more informative than the study of gene expression in nervous tissue, which includes a number of cell populations.

## 5. Conclusions

Thus, we have shown that the expression of genes encoding receptors for neurotransmitters, neuropeptides and hormones in DNs of the SNpc and in SNpc tissue differs significantly. In fact, *Gria2*, *Chrnb2*, *Gper1*, and *Igf1r* are predominantly expressed in DNs of the SNpc, whereas *Drd2*, *Grin2b*, *Grm1*, and *Ntsr2* expression predominates in SNpc tissue. In PD modeling, changes in the expression of genes encoding dopamine, glutamate and NT receptors are observed in DNs of the SNpc, but not in SNpc tissue. This means that studying gene expression in individual populations of SNpc cells is much more informative than an overall assessment of gene expression in different SNpc cells. Meanwhile, we note that changes in mRNA do not always correlate linearly with changes in functional protein levels due to post-transcriptional and post-translational regulation. Therefore, future studies should be aimed at precise evaluation of the synthesis and functional activity of the studied receptors in the norm and in PD modeling. This opens up broad prospects for the development of PD treatment with either agonists or antagonists of DNs receptors.

## Figures and Tables

**Table 1:** Oligonucleotide primers used for quantitative polymerase chain reaction.

Gene *	Protein	Forward Primer	Reverse Primer
*Cyc1*	Cytochrome C1	GCGGCCAGGGAAGTTGT	GCCAGTGAGCAGGGAAAATAC
*Drd2*	Dopamine receptor 2	GAACAGGCGGAGAATGGA	GGATGGATCGGGGAGAGT
*Grin2b*	Glutamate ionotropic receptor NMDA, subunit 2B	CGGCAGCACTCCTACGAC	CCAGCTGGCATCTCAAACATA
*Grin2d*	Glutamate ionotropic receptor NMDA, subunit 2D	TCCGCCTCAAGTACCCTCTAT	AAACCCTTGCAGCATCTCTTC
*Gria2*	Glutamate ionotropic receptor AMPA, subunit 2	GGGCGCTGATCAAGAATACA	TAAAACCCAAAAATCGCATAGAC
*Grm1*	Glutamate metabotropic receptor 1	ATGAACAAAAGCGGAATGGTA	CTCGAGGTAACGGATAGTAATGG
*Gabra3*	Gamma-aminobutyric acid type A receptor subunit alpha3	GCCGTCTGTTATGCCTTTGTAT	AGCAGCAGACTTGGAGATGGT
*Chrnb2*	Cholinergic receptor nicotinic beta 2 subunit	CTACACCATCAACCTCATCATCC	GCCAGCAGCACAGAAATACAA
*Cckar*	Cholecystokinin A receptor	AATAACCAGACGGCGAACAT	GTAAGCCACCACCATCACAAC
*Gper1*	G protein-coupled estrogen receptor 1	TCCGGGAGAAGATGACCA	GAGGAAGAAGACGCTGCTGTA
*Glp1r*	Glucagon-like peptide 1 receptor	CGGCGTCAACTTTCTTATCTTC	GGTTCCTCGGGCGTGTT
*Igf1r*	Insulin-like growth factor 1 receptor	CCGGACAACTGCCCTGATA	GGCTTGTTCTCCTCGCTGTAG
*Ntsr2*	Neurotensin receptor 2	GCGGTTATCATGGGACAGA	AAGGGGAGAACGAAGGAGA

* The gene nomenclature was taken from the National Library of Medicine’s GenBank database (https://www.ncbi.nlm.nih.gov/genbank (accessed on 15 September 2025)).

## Data Availability

Data were available on reasonable request to corresponding author.

## Abbreviations

The following abbreviations are used in this manuscript:

AMPA	α-amino-3-hydroxy-5-methyl-4-isoxazolepropionic acid
DNs	Dopaminergic neurons
GABA	Gamma-aminobutyric acid
GPER1	G protein-coupled estrogen receptor 1
IGF1	Insulin-like growth factor 1
mGluRs	Glutamate metabotropic receptors
MPTP	1-methyl-4-phenyl-1,2,3,6-tetrahydropyridine
nAChRs	Nicotinic acetylcholine receptors
NMDA	N-methyl-D-aspartate
NT	Neurotensin
PD	Parkinson’s disease
qPCR	Quantitative polymerase chain reaction
SNpc	Substantia nigra pars compacta
